# Ambiguity preferences for health

**DOI:** 10.1002/hec.3795

**Published:** 2018-07-03

**Authors:** Arthur E. Attema, Han Bleichrodt, Olivier L'Haridon

**Affiliations:** ^1^ Erasmus School of Health Policy & Management Erasmus University Rotterdam Rotterdam The Netherlands; ^2^ Erasmus School of Economics & Department of Health Policy & Management Erasmus University Rotterdam Rotterdam The Netherlands; ^3^ Research School of Economics Australian National University Canberra ACT Australia; ^4^ University of Rennes 1 Rennes France

**Keywords:** ambiguity, health

## Abstract

In most medical decisions, probabilities are ambiguous and not objectively known. Empirical evidence suggests that people's preferences are affected by ambiguity. Health economic analyses generally ignore ambiguity preferences and assume that they are the same as preferences under risk. We show how health preferences can be measured under ambiguity, and we compare them with health preferences under risk. We assume a general ambiguity model that includes many of the ambiguity models that have been proposed in the literature. For health gains, ambiguity preferences and risk preferences were indeed the same. For health losses, they differed with subjects being more pessimistic in decision under ambiguity. Utility and loss aversion were the same for risk and ambiguity. Our results imply that reducing the clinical ambiguity of health losses has more impact than reducing the ambiguity of health gains, that utilities elicited with known probabilities may not carry over to an ambiguous setting, and that ambiguity aversion may impact value of information analyses if losses are involved. These findings are highly relevant for medical decision making, because most medical interventions involve losses.

## 1 INTRODUCTION

Ambiguity is common in health decision making. The recent emphasis on evidence‐based medicine has highlighted that opinions conflict and that evidence is generally ambiguous. Ambiguity permeates not only clinical decision making but also other health decisions such as the adoption of healthy lifestyles, the choice of an insurance scheme, and public health decisions.

In the early 1990s, Pauker and Kopelman wrote a series of articles in the *New England Journal of Medicine* about cases in clinical decision making (e.g., Pauker & Kopelman, 1992a, 1992b, 1993, 1994a, 1994b). A common theme of these cases is ambiguity about the correct diagnosis. The case studies are full of words as “likely,” “maybe,” “I don't expect,” “I am not aware,” and so forth. Usually, several illnesses are possible and all the clinician can do is to assess their likelihood. Objective probabilities are never available.

In spite of the ubiquity of ambiguity in health, the question how people make health decisions under ambiguity has been largely ignored. There is a rich literature on health decision making under risk (where probabilities are objectively known), but very little attention has been paid to the arguably more realistic case where objective probabilistic information is missing. The most common approach is to assume that ambiguous prospects are treated similarly as risky prospects by replacing objective probabilities by the decision maker's subjective beliefs. This approach implicitly assumes that the decision maker is neutral towards ambiguity.

**Box 1. Introduction to ambiguity**

In decision making under ambiguity, outcomes are uncertain and their probabilities are unknown. There are several states of the world only one of which is true, but the decision maker does not know which one. A decision, or act, is a mapping that assigns an outcome to each state of the world. Under subjective expected utility (SEU), the decision maker evaluates an act *f* by its expected utility:

$$V_{\mathrm{EU}}\left(f\right)=E_{q}\left(u\left(f\right)\right)$$

where *u* is the utility function that is the same as the utility function under risk and, consequently, captures attitudes towards risk and *E*_*q*_ is the expectation with respect to the subjective probability measure *q* representing the decision maker's beliefs. Under SEU, attitudes towards uncertainty are entirely captured by selecting one single subjective probability measure *q* and the decision maker is ambiguity neutral.

SEU has been challenged by Ellsberg's (1961) paradox. Ellsberg conjectured that people would prefer a known urn containing 50 red and 50 black balls to an unknown urn containing also 100 red and black balls but in an unknown proportion both when red is the winning color and when black is the winning color. These preferences cannot be explained by a unique subjective probability measure as they imply that the decision maker's subjective probability of drawing a red ball and his subjective probability of drawing a black ball are both less than 0.5. Ellsberg's paradox suggests that people prefer decision situations with known probabilities to decision situations under ambiguity in which probabilities are unknown. This behavioral trait, called ambiguity aversion, has been confirmed in many experiments (Camerer & Weber, 1992). Later studies have shown that ambiguity aversion is not universal (Kocher, Lahno, & Trautmann, 2018): People are generally ambiguity seeking for losses and for unlikely events involving gains. Later research has also shown that ambiguity attitudes depend on the source of uncertainty. For example, people tend to be more ambiguity seeking for sources of ambiguity for which they consider themselves competent or that they are familiar with.

Many ambiguity models have been proposed to explain the Ellsberg paradox (see Etner, Jeleva, & Tallon, 2012, or Gilboa & Marinacci, 2016, for detailed surveys). These models can be subdivided into two classes.

A first class of ambiguity models models ambiguity aversion through a difference in utility between risk and uncertainty. We refer to this class of models as *source‐dependent utility* (SDU). The most popular SDU model is the “smooth model of ambiguity aversion” introduced by Klibanoff, Marinacci, and Mukerji (2005) and increasingly popular in economic applications. Other models in this class were proposed by Nau (2006), Ergin and Gul (2009), Seo (2009), and Neilson (2010).

A second class of ambiguity models models ambiguity aversion through a difference in decision weights for risk and ambiguity. We refer to this class as the *source‐dependent weighting* (SDW) models. Important examples are maxmin expected utility in which a decision maker has a set of possible beliefs and evaluates acts by the belief that gives the lowest expected utility, the less pessimistic *α*‐maxmin model that is a linear combination of maxmin expected utility and maxmax expected utility in which the acts are evaluated by the belief that gives the highest expected utility, and prospect theory in which subjective probabilities are replaced by decision weights that can be nonadditive. Other models belonging to this class include variational preferences (Maccheroni, Marinacci, & Rustichini, 2006), contraction expected utility (Gajdos, Hayashi, Tallon, & Vergnaud, 2008), vector‐expected utility (Siniscalchi, 2009), multiplier preferences (Hansen & Sargent, 2001; Strzalecki, 2011), and Choquet expected utility (Gilboa, 1987; Schmeidler, 1989; Wakker, 1987).

Ambiguity aversion may have important implications for the health domain. Many therapeutic decisions often involve uncertain probabilities, especially for treatments that are new or not used extensively, such as expensive treatments for cancer or other life‐threatening diseases. Ambiguity aversion implies that physician and government agencies will be reluctant to adopt new treatments that involve a lot of uncertainty (Viscusi & Zeckhauser, 2015). Moreover, health economic evaluations involve the modeling of uncertainty, for example, with Monte Carlo simulation or value of information (VoI) analysis (Claxton, 1999; Hoy, Peter, & Richter, 2014). Ambiguity models are particularly useful to capture such model uncertainty (see Maccheroni et al., 2006, or Strzalecki, 2011). Third, ambiguity preferences are relevant for lifetime health decisions, such as exercising and quitting smoking, which are known to reduce the probability of illness, but for which no exact probabilities exist.

The assumption of ambiguity neutrality is questionable. Keynes (1921) already pointed out that people's preferences over ambiguous prospects depend not only on their subjective beliefs but also on the confidence they have in those beliefs. Ellsberg's (1961) famous paradox showed that people usually prefer situations with known risks to situations with unknown risks. Empirical studies have confirmed such ambiguity aversion. Most of this evidence involves money, and there is little evidence on ambiguity and health. An exception is Curley, Eraker, and Yates (1984) who found ambiguity aversion for health, which differed from ambiguity aversion for money. This domain specificity of ambiguity preferences is consistent with studies on risk and time preferences which found that findings for money cannot be directly translated to health (Attema, Bleichrodt, L'Haridon, Peretti‐Watel, & Seror, 2018; Chapman, 1996; Hardisty & Weber, 2009; Weber, Blais, & Betz, 2002). Consequently, we cannot simply assume that ambiguity preferences for health mirror those for money.

Many models have been proposed to explain ambiguity aversion (Gilboa & Marinacci, 2016). Box 1 gives a brief introduction to ambiguity preferences and illustrates some applications to the health domain. As explained in Box 1, these models can be subdivided into two classes. The first class explains ambiguity aversion through a difference in utility between risk (known probabilities) and ambiguity (unknown probabilities). We refer to this class as the SDU class. The best known SDU model is the smooth ambiguity model of Klibanoff et al. (2005). The second class explains ambiguity aversion through a difference in the weighting of events under risk and ambiguity. We refer to this class as the SDW class. A popular SDW model is prospect theory (Kahneman & Tversky, 1979; Tversky & Kahneman, 1992). The empirical literature is divided as to which of these models best describes people's ambiguity preferences (Baillon & Bleichrodt, 2015; Cubitt et al., 2017; Chew, Miao, & Zhong, 2017). Cubitt et al. (2017) find that the smooth ambiguity model explains their subjects' ambiguity preferences better than *α*‐maxmin, an SDW model. Chew et al. (2017) interpret their data as supporting the model of Ergin and Gul (2009), an SDU model. On the other hand, the data in Baillon and Bleichrodt (2015) are most consistent with two SDW models, prospect theory and, to a lesser extent, *α*‐maxmin. They observed a fourfold pattern of ambiguity attitudes with ambiguity aversion for likely gains and unlikely losses and ambiguity seeking for unlikely gains and likely losses, which only prospect theory could fully explain. The few theoretical applications of ambiguity aversion to health have mostly used the smooth model (Berger, Bleichrodt, & Eeckhoudt, 2013; Berger, Emmerling, & Tavoni, 2016; Etner & Spaeter, 2010; Hoy et al., 2014; Treich, 2010), but empirical evidence supporting that people indeed behave according to the smooth model does not exist for health. 1On the other hand, Asano and Shibata (2011) use Gilboa and Schmeidler's (1989) maxmin expected utility model and Anwar and Zheng (2012) use Choquet expected utility.


This paper investigates in detail people's ambiguity preferences for health. Because ambiguity attitudes are usually sign dependent (Baillon & Bleichrodt, 2015, Trautmann and van de Kuilen, 2015), we consider both gains and losses in health. We assume a very general model of decision under ambiguity that includes most of the ambiguity models that have been proposed in the literature as special cases, and we show how this general model can be measured. We measure utility (including loss aversion) and event weights for health gains and losses for both risk and ambiguity. This allows us to answer the question whether ambiguity and risk preferences for health differ and whether the common approach in health economics to equate the two is justified. It also allows drawing some inferences about the descriptive validity of the SDU and SDW ambiguity models in health.

## 2 BACKGROUND

A decision maker has to make a choice under ambiguity. Ambiguity is modeled through a set of states of the worlds *S*. Exactly one of the states will obtain, but the decision maker does not know which one. Subsets *E* of *S* are called *events*, and *E*^*c*^ denotes the complement of *E*.

The decision maker has preferences over *health prospects* involving life duration. These preferences are denoted by the symbols ≻, ≽, and ~, which stand for strict preference, weak preference, and indifference, respectively. Preferences are defined relative to a reference point *x*_0_. *Gains* are outcomes strictly preferred to *x*_0_, and *losses* are outcomes strictly less preferred than *x*_0_. Health prospects are denoted *x*_*E*_*y*, signifying that the decision maker lives for *x* + *x*_0_ years if event *E* occurs and for *y* + *x*_0_ years otherwise. We assume that the decision maker prefers more life years to less. This excludes health states worse than death and health states for which there is a maximal endurable time (Stalmeier, Lamers, Busschbach, & Krabbe, 2007). If probabilities are known, we will write *x*_*p*_*y* for the prospect that gives life duration *x* + *x*_0_ years with probability *p* and life duration *y* + *x*_0_ years with probability 1 − *p*. We will refer to *x*_*E*_*y* as an *ambiguous prospect* (meaning that probabilities are unknown) and to *x*_*p*_*y* as a *risky prospect* (meaning that probabilities are known).

A prospect is *mixed* if it involves both a gain and a loss. For mixed prospects, the notation *x*_*E*_*y* signifies that *x* is a gain and *y* is a loss. A *gain prospect* involves no losses (i.e., both *x* and *y* are weakly preferred to *x*_0_) and a *loss prospect* involves no gains. For gain prospects, the notation *x*_*E*_*y* signifies that *x* ≥ *y*, and for loss prospects, it signifies that *x* ≤ *y*.

We assume that the decision maker evaluates mixed prospects *x*_*E*_*y* as

(1a) $$W^{+}\left(E\right)U\left(x\right)+W^{-}\left(E^{c}\right)U\left(y\right),$$

and gain or loss prospects as

(1b) $$W^{i}\left(E\right)U\left(x\right)+\left(1-W^{i}\left(E\right)\right)U\left(y\right),$$

where *i* = + for gains and *i* = − for losses. *U* is a strictly increasing, real‐valued *utility function* that satisfies *U*(*x*_0_) = 0. The utility function is a ratio scale, and we can choose the utility of one outcome other than the reference point. *U* is an overall utility function that includes loss aversion.

The *event weighting functions* *W*^*i*^, *i* =  + , −, assign a number *W*^*i*^(*E*) to each event *E* such that

1. *W*^*i*^(∅) = 0.
2. *W*^*i*^(*S*) = 1.
3. *W*^*i*^ is *monotonic*: *E* ⊇ *F* implies *W*^*i*^(*E*) ≥ *W*^*i*^(*F*).

The event weighting functions *W*^*i*^ may be different for gains and losses, and they need not be additive. If they are additive, the event weights are subjective probabilities and Equations 1a and 1b are equivalent to SEU.

The model described in Equations 1a and 1b is referred to in the literature as *biseparable preferences* (Ghirardato & Marinacci, 2001). It is very general and includes many of the ambiguity models that have been proposed in the literature as special cases (Wakker, 2010). For that reason, we take it as our structural assumption and measure its distinct components.

Under biseparable preferences, mixed risky prospects *x*_*p*_*y* are evaluated as

(2a) $$w^{+}\left(p\right)u\left(x\right)+w^{-}\left(1-p\right)u\left(y\right),$$

and gain and loss risky prospects *x*_*p*_*y* as

(2b) $$w^{i}\left(p\right)u\left(x\right)+\left(1-w^{i}\left(p\right)\right)u\left(y\right),i=+,-.$$

*w*^*i*^ is a strictly increasing *probability weighting function* that satisfies *w*^*i*^(0) = 0 and *w*^*i*^(1) = 1 and that may also differ between gains and losses. *u* is a strictly increasing real‐valued utility function that satisfies *u*(*x*_0_) = 0. Hence, in the evaluation of risky prospects, the event weighting functions *W*^*i*^ are replaced by probability weighting functions *w*^*i*^ and the utility function *U* is replaced by *u*.

By comparing utility and event weighting under risk and ambiguity, we can evaluate whether preferences under risk can be used to inform preferences under ambiguity. This comparison also allows us to test the descriptive validity of the SDU and the SDW models for health. The SDU models assume that *W*^*i*^ = *w*^*i*^ and they model ambiguity aversion by a difference between *U* and *u*. More precisely, the decision maker is ambiguity averse (seeking) in the SDU models if *U* is a concave (convex) transformation of *u*. On the other hand, the SDW models assume that *U* = *u* and they model ambiguity aversion by means of a difference between *W*^*i*^ and *w*^*i*^. In the SDW models, ambiguity aversion (seeking) for gains means that *W*^+^ lies below (above) *w*^+^, and ambiguity aversion (seeking) for losses means that *W*^−^ lies above (below) *w*^−^. Box 2 describes how we measured utility and event weighting for ambiguity and risk.

**Box 2. Measurement method**

We used the method of Abdellaoui, Bleichrodt, L'Haridon, and van Dolder (2016) to measure *U* and *u* in Equations 1a and 1b and 2a and 2b. By adding a few questions, we could also measure *W*^*i*^ and *w*^*i*^, *i* =  + , −. We imposed no simplifying parametric assumptions on utility, loss aversion, probability weighting, or event weighting. Consequently, our measurements are entirely parameter free.

The measurements were performed in four stages, which are summarized in Table 1. The first three stages measured utility for gains and losses, and the fourth stage measured event/probability weighting. We will describe the measurement procedure for ambiguity. The measurements for risk follow by replacing the event *E* by a given probability *p*. The third column of Table 1 shows the quantity that was assessed in each of the four stages of the procedure. The fourth column shows the indifference that was elicited. The fifth column shows the implication of the elicited indifference. The sixth column shows the stimuli that we used in the experiment reported in Section 4.

The first stage established the link between utility for gains and utility for losses by eliciting a gain and a loss with the same absolute utility. We started by selecting an event *E* and a gain *G*. Then we elicited the loss *L* for which *G*_*E*_*L*~*x*_0_ and certainty equivalents $x_{1}^{+}$ and $x_{1}^{-}$ such that $x_{1}^{+}\sim G_{E}x_{0}$ and $x_{1}^{-}\sim L_{E^{c}}x_{0}$. Abdellaoui et al. (2016) showed that these three indifferences imply that

(3) $$U\left(x_{1}^{+}\right)=-U\left(x_{1}^{-}\right).$$

In other words, $x_{1}^{+}$ and $x_{1}^{-}$ are a gain and a loss that have the same absolute utility.

In the second stage, we used $x_{1}^{+}$ and the trade‐off method of Wakker and Deneffe (1996) to elicit a sequence of gains $x_{2}^{+},\ldots ,x_{5}^{+}$ for which the utility difference between successive elements was constant. Let *ℓ* be a prespecified loss. We first elicited the loss L such that the subject was indifferent between ${x_{1}^{+}}_{E}L$ and $\ell_{E^{c}}x_{0}$. This established a gauge that we used next to elicit a series of indifferences ${x_{j}^{+}}_{E}L\sim {x_{j-1}^{+}}_{E}\ell$, *j* = 2, …, 5. Wakker and Deneffe (1996) showed that the utility difference between the successive elements of the sequence $x_{0},x_{1}^{+},\ldots ,x_{5}^{+}$ is constant: $U\left(x_{j}^{+}\right)-U\left(x_{j-1}^{+}\right)=U\left(x_{1}^{+}\right)-U\left(x_{0}\right)$, *j* = 2, …, 5.

The third stage was similar to the second except that we used $x_{1}^{-}$ to construct a sequence of losses $x_{0},x_{1}^{-},\ldots ,x_{5}^{-}$ for which the utility difference between successive elements was constant. We selected a gain *ℊ* and an event *E* and elicited the gain G such that $G_{E}x_{1}^{-}\sim {ℊ}_{E}x_{0}$. We then proceeded to elicit the sequence $\left\{x_{0},x_{1}^{-},x_{2}^{-},\ldots ,x_{k_{L}}^{-}\right\}$ by eliciting a series of indifferences $G_{E}x_{j}^{-}\sim {ℊ}_{E}x_{j-1}^{-}$, *j* = 2, …, *k*_*L*_.

Because $U\left(x_{1}^{+}\right)=U\left(x_{1}^{-}\right)$, the second‐stage and third‐stage sequences could be combined to obtain a sequence $\left\{x_{5}^{-},\ldots ,x_{1}^{-},x_{0},x_{1}^{+},\ldots ,x_{5}^{+}\right\}$ that ran from the domain of losses through the reference point to the domain of gains and for which the utility difference between successive elements was constant. We scaled utility by setting $U\left(x_{5}^{+}\right)=1$, which is allowed by the uniqueness properties of biseparable preferences. It follows that $U\left(x_{j}^{+}\right)=j/5$ and $U\left(x_{j}^{-}\right)=-j/5$, for *j* = 1, …, 5.

In the fourth stage, we used the elicited sequence $\left\{x_{5}^{-},\ldots ,x_{1}^{-},x_{0},x_{1}^{+},\ldots ,x_{5}^{+}\right\}$ to measure the probability and the event weights. For an event *E*, we measured *W*^+^(*E*) by eliciting the certainty equivalent $x_{E}^{+}$ of the prospect $\left(x_{5}^{+},E,x_{0}\right)$. Then $U\left(x_{E}^{+}\right)=W^{+}\left(E\right)$. $U\left(x_{E}^{+}\right)$ can be approximated from the utility function for ambiguity that was measured in the second stage. Similarly, we measured *W*^−^(*E*) by eliciting the certainty equivalent $x_{E}^{-}$ of the prospect $\left(x_{5}^{-},E,x_{0}\right)$. We varied *E* to measure 5 points of *W*^*i*^, *i* =  + , −. Similarly, we used five probabilities to measure the probability weighting functions *w*^*i*^, *i* =  + , −.

## 3 EXPERIMENT

### 3.1 Subjects

Subjects were 65 students of the Erasmus University (27 female). They were recruited from the subject pool of the ESE lab by means of the ORSEE recruiting system (Greiner, 2015). The average age of the participant was 21.5 years, with a standard deviation of 1.9 years. Each subject was paid a €10 participation fee. No other incentives could be implemented because we used health stimuli. Data were collected by individual interviews to improve data quality. The experiment was computer run. Subjects first received instructions about the tasks. They were told that there were no right or wrong answers and that we were only interested in their preferences. We emphasized that they should go through the experiment at their own pace. After the instructions, subjects completed 10 practice questions. Then they started with the actual experiment. All 65 subjects completed the experiment. The experiment satisfied the ethical guidelines of the ESE lab, and no further ethical approval was needed. The experimental instructions are in Appendix S1. 2Prior to the actual experiment, we did an extensive pilot study, which mainly served to fine‐tune the implementation of the ambiguity questions.


### 3.2 Procedure and stimuli

Table 1 shows the values of the parameters that we specified in advance. We told subjects to imagine living with a disease that restricted their life expectancy to 50 more years, but which did not affect their quality of life. These 50 years are about 10 years below the life expectancy of the average subject in our sample. The disease required taking a drug with no side effects. If the subject would not take a drug, he would die immediately. Subjects were asked to choose between two drugs that had two possible outcomes. Under risk, the success rates of the drugs were objectively given. Under ambiguity, we specified a range of possible success rates. 3This implementation of ambiguity was similar to Curley et al. (1984) and Curley and Yates (1985).


**Table 1:** The four‐stage measurement method

		Assessed quantity	Indifference	Implication	Stimuli
Stage 1	Mixed prospect followed by two certainty equivalents	*L*	*G*_*E*_*L*~0	$U\left(x_{1}^{+}\right)=-U\left(x_{1}^{-}\right)$	*G* = 32 months Unc.: *E* = [0.3,0.7] Risk: *p* = ½
		$x_{1}^{+}$	$x_{1}^{+}\sim G_{E}0$		
		$x_{1}^{-}$	$x_{1}^{-}\sim L_{E^{c}}0$		
Stage 2	Step 1: Elicitation of gauge value L	L	${x_{1}^{+}}_{E}L\sim \ell_{E^{c}}0$	$U\left(x_{j}^{+}\right)-U\left(x_{j-1}^{+}\right)=U\left(x_{1}^{+}\right)-U\left(x_{0}\right)$	*ℓ* = − 6 months j = 2, …, 5
	Steps 2 to 5: Standard sequence for gains	$x_{j}^{+}$	${x_{j}^{+}}_{E}L\sim {x_{j-1}^{+}}_{E^{c}}\ell$		
Stage 3	Step 1: Elicitation of gauge value G	G	$G_{E}x_{1}^{-}\sim {ℊ}_{E}0$	$U\left(x_{j}^{-}\right)-U\left(x_{j-1}^{-}\right)=U\left(x_{1}^{-}\right)-U\left(x_{0}\right)$	*ℊ* = 6 months j = 2, …, 5
	Steps 2 to 5: Standard sequence for losses	$x_{j}^{-}$	$G_{E}x_{j}^{-}\sim {ℊ}_{E}x_{j-1}^{-}$		
Stage 4: Certainty equivalents to elicit event/probability weights	Certainty equivalents for gains	$x_{E}^{+}\;$	$x_{E}^{+}\sim {x_{5}^{+}}_{E}0$	$U\left(x_{E}^{+}\right)=W^{+}\left(E\right)$	Unc.: *E* = [0,0.2], [0.1,0.5], [0.3,0.7], [0.5,0.9], [0.8,1] Risk: *p* = 0.1, 0.3, 0.5, 0.7, 0.9
	Certainty equivalents for losses	$x_{E}^{-}\;$	$x_{E}^{-}\sim {x_{5}^{-}}_{E}0$	$U\left(x_{E}^{-}\right)=W^{-}\left(E\right)$	

**Figure 1:**
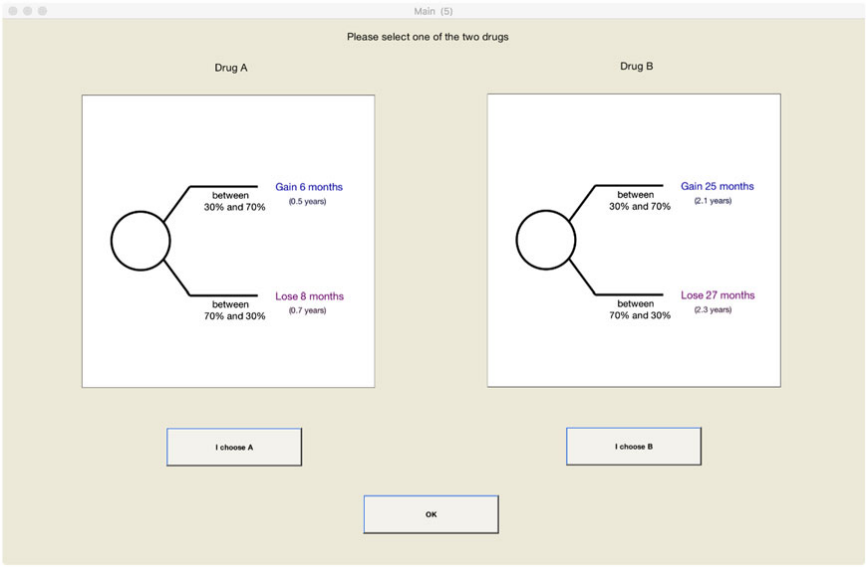
Presentation of the choices under ambiguity [Colour figure can be viewed at http://wileyonlinelibrary.com]

The outcomes of the two drugs were described as gains and losses in life expectancy from 50 years. By presenting the choices this way, we hoped that subjects would take 50 years as their reference point. This strategy has been successfully applied before by Attema, Brouwer, and Haridon (2013). Figure 1 shows the presentation of the choices under ambiguity. The presentation under risk was similar except that the success rates were objectively given.

For both gains and losses, we elicited 5 points of the utility function under both risk and ambiguity. For risk, we elicited the weights of five probabilities for both gains and losses: 0.1, 0.3, 0.5, 0.7, and 0.9. These include a probability that is usually overweighted (0.1), two probabilities that are usually underweighted (0.7 and 0.9), and two probabilities for which usually little weighting is observed (0.3 and 0.5; Fox & Poldrack, 2014). For ambiguity, we presented subjects with an interval within which the imprecise success rates could lie. The centers of these intervals were equal to the success rates considered for risk. Their ranges were [0, 0.2], [0.1, 0.5], [0.3, 0.7], [0.5, 0.9], and [0.8, 1]. So for the smallest and largest success rates, the range of possible probabilities was 0.2; for the other probabilities; it was 0.4. Curley and Yates (1985) found no effect of the (nonzero) range of probabilities on ambiguity attitudes.

We used a choice‐based procedure to elicit indifferences. The procedure zoomed in on subjects' indifference values by an iterative series of binary choices. Previous research suggests that choice‐based elicitation leads to more reliable results than asking subjects directly for their indifference values (Bostic, Herrnstein, & Luce, 1990).

The iterative procedure used five choices on average. If the interval in which the indifference value fell became less than a month, the process stopped. At the end of the bisection process, the program asked subjects to confirm their choice. If so, they moved on to the next elicitation. If not, the process for that elicitation started anew. In the analyses, we used the indifference value for which subjects confirmed their choice.

We randomized the order of the risk and the ambiguity parts. When a subject had completed the first part, the interviewer would point out the differences with the next part of the experiment before proceeding. Within the risk and ambiguity parts, we randomized the order of the second (the elicitation of the utility for gains) and the third stages (the elicitation of the utility for losses) of the measurement procedure. The first stage always had to come first, because it produced the inputs for the second and third stages. The fourth stage always came last because it required information from the second and third stages. Within the fourth stage, we also randomized whether the gain or the loss part came first.

To test for consistency and to obtain insight into the quality of the data, we included two types of repetitions. First, we repeated the third iteration of the bisection process in 12 tasks. In the third iteration, most subjects were close to indifference and, hence, this was a rather strong test of consistency. Second, at the end of the second stage, the elicitation of the gain sequence, we repeated the elicitation of $x_{3}^{+}$, both in the risk and in the ambiguity part.

Box 3 describes the analyses we performed.

**Box 3. Analyses**

Utility curvature

We used two different methods to investigate utility curvature, one nonparametric and the other parametric. The nonparametric method calculated the area under the utility function. The domain of *U* was normalized to [0, 1] by transforming every gain $x_{j}^{+}$ to $x_{j}^{+}/x_{5}^{+}$ and every loss $x_{j}^{-}$ to $x_{j}^{-}/x_{5}^{-}$. If utility is linear, the area under the normalized curve equals ½. For gains, utility is convex (concave) if the area under the curve is smaller (larger) than ½. For losses, utility is convex (concave) if the area under the curve is larger (smaller) than ½.

In the parametric method, we estimated the utility function by the power family, the most commonly employed parametric family (Wakker, 2008). The power family is defined by *x*^*α*^ with *α* > 0. For gains (losses), *α* > 1 corresponds to convex (concave) utility, *α* = 1 corresponds to linear utility, and *α* < 1 corresponds to concave (convex) utility. Estimation was done by nonlinear least squares. As the results from the parametric estimation were similar to those of the nonparametric analysis, we will concentrate on the nonparametric results. The parametric results are reported in Appendix S1.

Loss aversion

To measure loss aversion, we used Kahneman and Tversky's (1979) definition of loss aversion. They define loss aversion as −*U*(−*x*) > *U*(*x*) for all *x* > 0. This definition reflects that losses loom larger than gains as the absolute utility of any loss exceeds the utility of the commensurate gain. To measure loss aversion coefficients, we computed $-U\left(-x_{j}^{+}\right)/U\left(x_{j}^{+}\right)$ and $-U\left(-x_{j}^{-}\right)/U\left(x_{j}^{-}\right)\;$for *j* = 1, …, 5, whenever possible. When $U\left(-x_{j}^{+}\right)$ and $U\left(-x_{j}^{-}\right)$ could not be observed directly, we estimated them through linear extrapolation using the elements of the elicited sequence $\left\{x_{5}^{-},\ldots ,x_{1}^{-},x_{0},x_{1}^{+},\ldots ,x_{5}^{+}\right\}$. A subject was classified as loss averse if −*U*(−*x*)/*U*(*x*) > 1 for all observations, as loss neutral if −*U*(−*x*)/*U*(*x*) = 1 for all observations, and as gain seeking if −*U*(−*x*)/*U*(*x*) < 1 for all observations. To account for response error, we also used a more lenient rule, which classified subjects as loss averse, loss neutral, or gain seeking if the above held for more than half of the observations.

To test for robustness, we also used Köbberling and Wakker's (2005) definition of loss aversion according to which a decision maker is loss averse if the kink of utility at the reference point exceeds 1. They define an index of loss aversion as $U_{↑}^{\prime }\left(0\right)/U_{↓}^{\prime }\left(0\right)$, where $U_{↑}^{\prime }\left(0\right)$ represents the left derivative and $U_{↓}^{\prime }\left(0\right)$ represents the right derivative of *U* at the reference point. In our method, this definition is measured by the ratio $x_{1}^{+}/-x_{1}^{-}$, which requires no interpolation of utility. A subject was classified as loss averse if this ratio exceeded one, as loss neutral if it was equal to one, and as gain seeking if it was smaller than one. Statistical testing confirmed that the classification of the subjects and the loss aversion coefficients were similar to those under the definition of Kahneman and Tversky (1979) and, consequently, all conclusions were the same (see Appendix S1 for details).

Probability weighting and event weighting

To measure probability and event weighting requires knowledge of $U\left(x_{p}^{+}\right)$, $U\left(x_{p}^{-}\right)$, $U\left(x_{E}^{+}\right)$, and $U\left(x_{E}^{-}\right)$ for *p* = 0.1, 0.3, 0.5, 0.7, 0.9 and *E* = [0, .2], [.1, .5], [.3, .7], [.5, .9], [.8,1]. We used linear interpolation to measure these utilities. To compare our results with those from the literature, we also performed a parametric estimation of the probability weighting function using Prelec's (1998) two‐parameter specification $w^{i}\left(p\right)=\exp\left\{-\delta^{i}{\left(-\mathrm{lnp}\right)}^{\gamma^{i}}\right\},i=+,-$. For ambiguity, we set *p* equal to the midpoint of the range of possible probabilities in this estimation. The *δ* parameter controls for pessimism with higher values corresponding with less pessimism. The *γ* parameter corresponds with sensitivity to changes in likelihood with higher values corresponding with higher sensitivity. Estimation was by nonlinear least squares. To test for robustness, we also used the neo‐additive weighting function of Chateauneuf, Eichberger, and Grant (2007). This analysis gave the same results and is reported in Appendix S1.

The question that we seek to address is whether utility and event weighting are the same for risk and ambiguity. Hence, our main interest is to test for equalities of subjective parameters. Classic significance tests are less suitable for this as they do not allow to state evidence for the null and they overstate the evidence against the null (Rouder, Speckman, Sun, Morey, & Iverson, 2009). We therefore used Bayesian statistics and Bayes factors instead. Bayes factors indicate how much more likely the alternative is than the null. For example, a Bayes factor of 10 indicates that the alternative is 10 times as likely as the null given the data. A Bayes factor of 0.10 indicates that the null is 10 times as likely as the alternative given the data. We used the common interpretation that a Bayes factor larger than 3 signals some support for the alternative over the null, a Bayes factor larger than 10 signals strong support for the alternative over the null, and a Bayes factor larger than 30 signals very strong support for the alternative over the null. Similarly, a Bayes factor less than 0.33 signals some support for the null over the alternative, a Bayes factor less than 0.10 signals strong support for the null over the alternative, and a Bayes factor less than 0.03 signals very strong support for the null over the alternative. To check for robustness and because the Bayesian *t* test is sensitive to the variance of the underlying distributions, we also performed classic nonparametric tests. These generally led to the same conclusions (unless otherwise stated) and are reported in Appendix S1.

## 4 RESULTS

### 4.1 Consistency checks

Subjects made the same choice in 76% of the repetitions of the third iteration of the bisection process as in the original choice. This is comparable to the reversal rates, which are commonly observed in the literature (Stott, 2006), especially if we take into account that subjects were close to indifference in the third iteration. A Bayesian analysis of variance (ANOVA) showed support for the null that consistency was the same for risk and ambiguity and for gains and losses (*BF* = 0.21).

**Figure 2:**
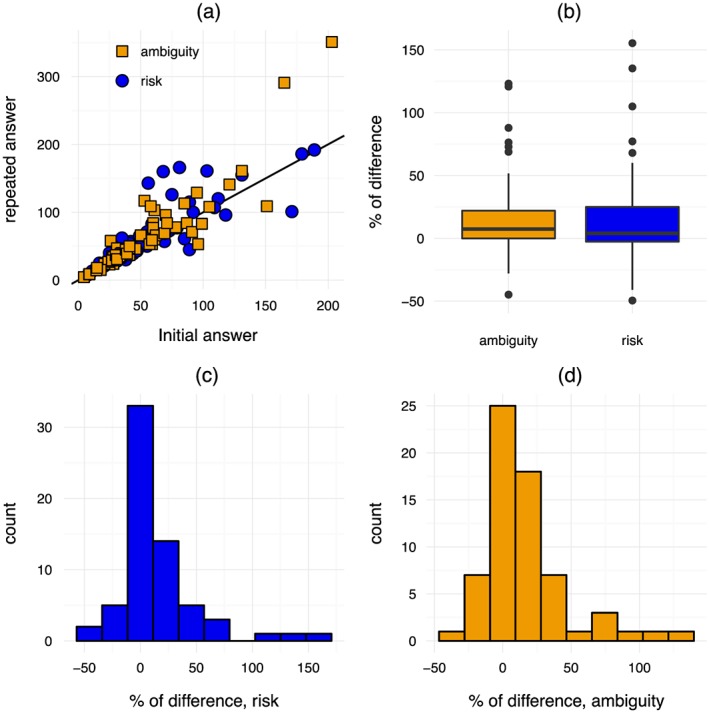
Original and repeated elicitation of the third indifference value for gains [Colour figure can be viewed at http://wileyonlinelibrary.com]

Figure 2 shows the results of the original and the repeated elicitation of $x_{3}^{+}$. Figure 2a shows that the two elicitations were closely related except for a few outliers. The correlation was almost perfect. For risk, the Spearman rank correlation was 0.92; for ambiguity, it was 0.93. Figure 2b shows that the difference between the original and the repeated elicitation as a percentage of the original elicitation was centered around zero. Figure 2c shows a histogram for the % difference for risk, and Figure 2d for ambiguity. These panels show a slight tendency for higher values in the repeated elicitation. For risk, a Bayesian analysis was inconclusive (*BF* = 1.25). For ambiguity, we found some support for the hypothesis that the original and the repeated elicitation indeed differed (*BF* = 4.34).

### 4.2 Ambiguity aversion

Ambiguity aversion means that subjects prefer decisions under risk to decisions under ambiguity when the objective and subjective probabilities are the same. The first two choices of our measurement method allowed two tests of ambiguity aversion. In the first choice, ambiguity aversion predicts that *L*_*a*_ > *L*_*r*_ where the subscripts *a* and *r* stand for ambiguity and risk, respectively. 4By ambiguity aversion, *G*_*p*_*L* ≻ *G*_*E*_*L* for any *G* and *L* if the subjective probability of event *E* is equal to *p*. Thus, 0~*G*_*p*_*L*_*r*_ ≻ *G*_*E*_*L*_*r*_, and thus, 0~*G*_*E*_*L*_*a*_ ≻ *G*_*E*_*L*_*r*_, which implies *L*_*a*_ > *L*_*r*_. In the second choice, ambiguity aversion predicts that $x_{1,r}^{+}>x_{1,a}^{+}$. 5By ambiguity aversion, *G*_*p*_0 ≻ *G*_*E*_0 and, thus, $x_{1,r}^{+}\sim G_{p}0≻G_{E}0\sim x_{1,a}^{+}$.


**Figure 3:**
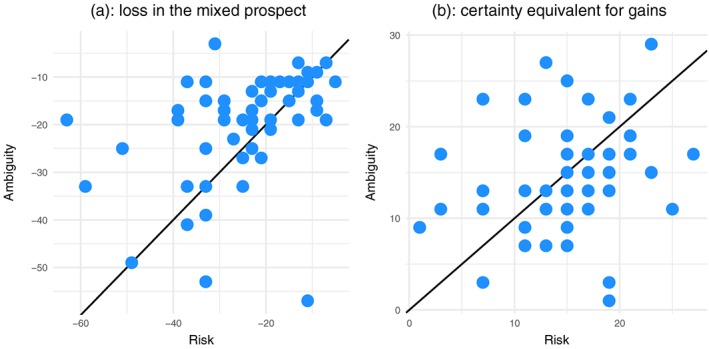
Tests of ambiguity aversion [Colour figure can be viewed at http://wileyonlinelibrary.com]

Figure 3a shows the relation between *L*_*r*_ and *L*_*a*_. The figure shows that most values of *L*_*a*_ were above the diagonal, which is consistent with ambiguity aversion. Statistical testing showed that the hypothesis that *L*_*a*_ > *L*_*r*_ was nearly 3 times as likely as the null that *L*_*a*_ = *L*_*r*_ (*BF* = 2.92, *p* < 0.01 in a Wilcoxon test). However, Figure 3b, which displays the relation between $x_{1,r}^{+}$ and $x_{1,a}^{+}$, shows no evidence for ambiguity aversion. Ambiguity aversion would here predict that the points lie below the diagonal, but there was no obvious pattern. Indeed, the data supported the null that $x_{1,r}^{+}=x_{1,a}^{+}$, that is, ambiguity neutrality, over the alternative of ambiguity aversion (*BF* = 0.14).

Table 2 shows the classification of the subjects. The table shows that ambiguity aversion was the most common pattern in both tests. For mixed prospects, we found support for ambiguity aversion (*BF* = 8.14). For gain prospects, the test was inconclusive (*BF* = 0.51).

**Table 2:** Classification of subjects in terms of ambiguity attitude

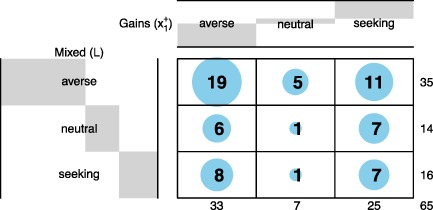

### 4.3 The utility for gains and losses

**Figure 4:**
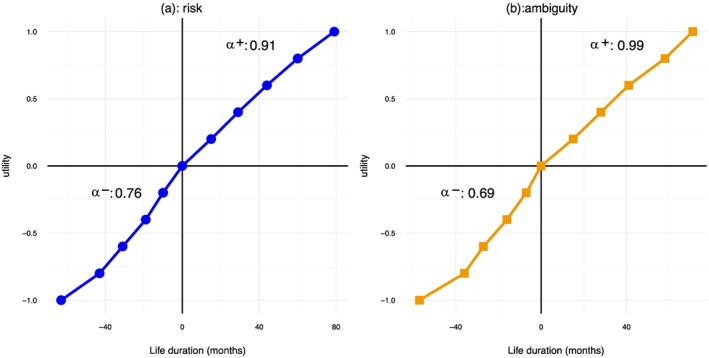
The utility for gains and losses based on the median data [Colour figure can be viewed at http://wileyonlinelibrary.com]

Figure 4 shows the utility for gains and losses under risk (Figure 4a) and ambiguity (Figure 4b) based on the median data. Two things are noteworthy. First, the utility functions under risk and ambiguity look similar, and second, they are consistent with the typical finding of convex utility for losses and concave utility for gains. For ambiguity, utility was close to linear for gains. Moreover, utility was more curved for losses than for gains. For money, most studies found the opposite pattern. The figure also shows the estimated power coefficients based on the median data.

**Figure 5:**
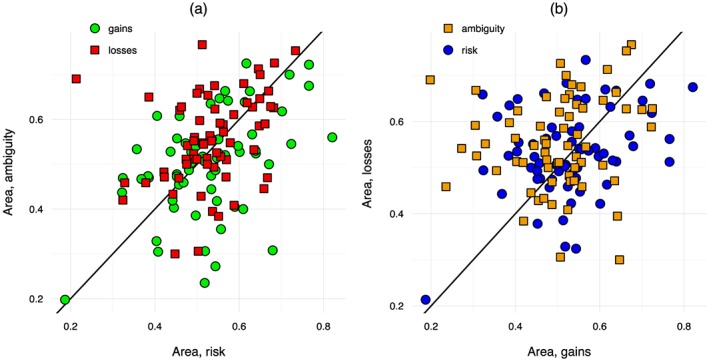
Individual shapes of utility [Colour figure can be viewed at http://wileyonlinelibrary.com]

Moving to the individual data, Figure 5 shows the relation between the area measures between risk and ambiguity (Figure 5a) and between gains and losses (Figure 5b). Figure 5a shows that there was no clear difference in the shape of utility between risk and ambiguity. Indeed, a Bayesian ANOVA supported the null that utility was the same for risk and ambiguity (*BF* = 0.14). Figure 5b shows that most points were above the diagonal signaling more curvature for losses than for gains. A Bayesian ANOVA showed that the hypothesis that utility was different for gains and losses was about 3 times as likely as the null that utility was the same (*BF* = 2.89). 6
*p* = 0.03 by a standard ANOVA.


Table 3 shows the classification of subjects according to the shape of their utility function. Table 3a gives the results for risk, and Table 3b those for ambiguity. The table confirms the impressions obtained above. The classification was similar for risk and ambiguity and the common pattern was S‐shaped utility: concave for gains and convex for losses. However, although statistical tests showed support for the hypothesis that utility was convex for losses (*BF* = 5.67 for risk and *BF* = 370.22 for ambiguity), we found no evidence that utility was concave for gains. The data were inconclusive as to the concavity of utility for risk (*BF* = 0.95), and they supported the null of linearity for ambiguity (*BF* = 0.14). Only a small minority of the subjects behaved according to the traditional assumption in economics that utility under risk is concave throughout. In fact, there were more subjects with everywhere convex utility. 7The parametric results confirmed that utility was the same for risk and ambiguity, but they showed less of a difference in utility curvature between gains and losses. They are reported in Appendix S1.


**Table 3:** Classification of subjects by the shape of their utility function

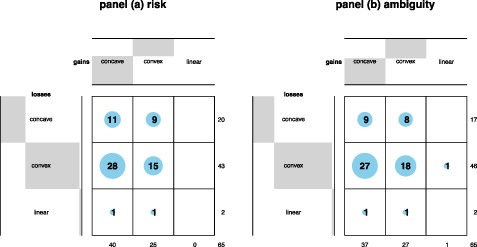

### 4.4 Loss aversion

The previous subsection showed little differences in utility curvature between risk and ambiguity. Consequently, to explain the ambiguity aversion that we observed for mixed prospects by differences in utility as the SDU class of ambiguity models does, loss aversion should differ between risk and ambiguity. We will now explore whether it did.

**Figure 6:**
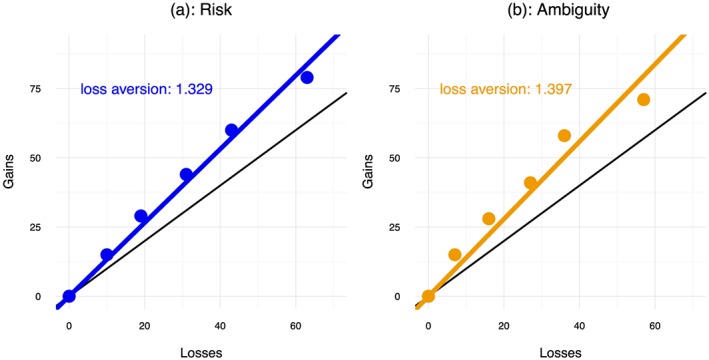
The relation between median gains and median losses with the same absolute utility [Colour figure can be viewed at http://wileyonlinelibrary.com]

Figure 6 displays the relations between the medians of $x_{j}^{+}$ and $-x_{j}^{-}$ under risk and under ambiguity. Consistent with Kahneman and Tversky's (1979) definition of loss aversion, $-x_{j}^{-}$ was always lower than $x_{j}^{+}$ (for all *j*) for both risk and ambiguity. We obtain an aggregate measure of loss aversion by regressing the $x_{j}^{+}$ on $\left(-x_{j}^{-}\right)$ in each panel. Figure 6 displays the coefficients from these regressions. The coefficients were close for risk and ambiguity. At the aggregate level, loss aversion was moderate and somewhat lower than what has typically been found for money.

Moving to the individual level, a Bayesian ANOVA showed strong support for the null that the ratios $x_{j}^{+}/-x_{j}^{-}$ were the same for risk and ambiguity (*BF* = 0.09). We also found very strong support that they were constant across tasks (*BF* = 0.00). The medians of the individual ratios varied between 1.45 and 1.92 for risk and between 1.50 and 1.75 for ambiguity. These values are comparable to the loss aversion coefficients found by Bleichrodt, Abellan‐Perpiñan, Pinto‐Prades, and Mendez‐Martinez (2007) for health. They are higher than those found by Attema et al. (2013).

Table 4 shows the classification of the subjects in terms of loss aversion based on Kahneman and Tversky's (1979) measure. There was clear evidence of loss aversion regardless of whether we used the strict rule that all choices should be consistent with a particular pattern or the more lenient rule, which allows for response error, that the majority of choices should be consistent with a particular pattern. We found very strong evidence that there were more loss averse than gain seeking subjects (both *BF* > 174.1). The median loss aversion coefficients were 1.45 for risk and 1.57 for ambiguity. We found support for the hypothesis that they differed from 1, the case of loss neutrality (*BF* = 13.02 for risk and *BF* = 7.39 for ambiguity). Most importantly, we found support for the null that loss aversion was the same for risk and ambiguity (*BF* = 0.14) signaling that loss aversion could not explain the ambiguity aversion for mixed prospects that we observed.

**Table 4:** Individual classification in terms of loss aversion for risk and ambiguity

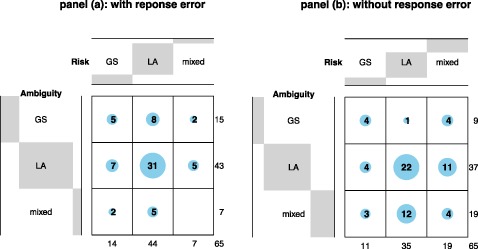

### 4.5 Probability weighting and event weighting

**Figure 7:**
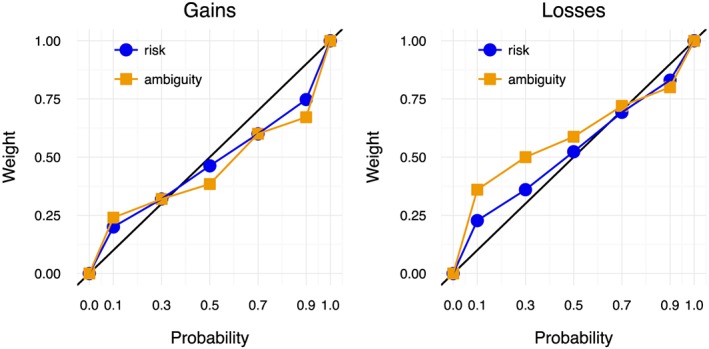
Probability and event weighting functions for gains and losses based on the median data [Colour figure can be viewed at http://wileyonlinelibrary.com]

Figure 7 shows the probability and event weighting functions for gains and losses based on the median data. Recall that the class of SDW ambiguity models explains ambiguity aversion by a difference between probability and event weighting.

For gains, the probability weighting and event weighting functions look similar except perhaps for probability 0.5. This is consistent with the absence of ambiguity aversion for gains that we observed. However, for losses, the event weighting function was more elevated than the probability weighting function for probabilities smaller than 0.75, which implies ambiguity aversion for losses. Identical probability and event weighting for gains, but higher event weighting than probability weighting for losses (in combination with the same utility curvature and loss aversion for risk and ambiguity that we observed above), can indeed explain the ambiguity aversion for mixed prospects that we observed.

A Bayesian ANOVA showed very strong evidence that probability and event weighting depended on the domain (gains vs. losses) and support for the hypothesis that the interaction between domain and context (risk vs. ambiguity) mattered (*BF* = 7.02). This is consistent with our above observations that probability and event weighting were similar for gains but differed for losses. Looking at the separate probabilities for gains, we found support for the null that probability weighting and event weighting of 0.1, 0.3, and 0.7 were the same (all *BF* < 0.19), whereas for probabilities 0.5 and 0.9, the evidence was inconclusive. For losses, we found very strong support for the hypothesis that the probability and event weight of 0.1 differed (*BF* = 45.14) and support that they differed for probability 0.3 (*BF* = 4.12). For probability 0.5, the evidence was inconclusive (*BF* = 0.93), and for probabilities 0.7 and 0.9, we found support for the null that the probability and event weight were the same (both *BF* < 0.20).

Both the probability weighting and the event weighting functions had an inverse S‐shape, as commonly observed in empirical research (Fox & Poldrack, 2014). This shape implies that unlikely events are overweighted and that more likely events are underweighted. We found very strong evidence that probability 0.1 was overweighted and that probability 0.9 was underweighted for both risk and ambiguity and for both gains and losses (all *BF* > 69,295). For gains, we also found very strong evidence that probability 0.7 was underweighted (both *BF* > 6,811). For losses, we found very strong evidence that probability 0.3 was overweighted (both *BF* > 93.8).

For the other cases, the results depended on the context and on the domain. For risk and gains, we found support that probability 0.3 was overweighted (*BF* = 6.11) and for the null that there was no probability weighting of probability 0.5 (*BF* = 0.30). The probability weighting for gains was comparable with Bleichrodt and Pinto (2000) who also found overweighting of probabilities less than 0.5, no probability weighting for probability 0.5, and underweighting of probabilities exceeding 0.5. For risk and losses, we also found support for the null of no weighting of probability 0.5 (*BF* = 0.21), but inconclusive evidence about the weighting of probability 0.7 (*BF* = 1.14).

For ambiguity and gains, the evidence was inconclusive regarding the weighting of probability 0.3 (*BF* = 2.13), but we found very strong evidence for the underweighting of probability 0.5 (*BF* = 42.5). For ambiguity and losses, we found very strong evidence that probability 0.5 was overweighted (*BF* = 32.4) and support for the null of no weighting of probability 0.7 (*BF* = 0.28).

As we mentioned above, the Bayesian ANOVA showed that the weights differed between gains and losses. For risk, we found evidence that the weights of probabilities 0.7 and 0.9 differed (both *BF* > 4.95). For probabilities 0.1 and 0.3, we found support for the null of no difference (both *BF* < 0.15), and for probability 0.5, the evidence was inconclusive (*BF* = 0.46). For ambiguity, we found very strong support that the weighting of probabilities 0.5, 0.7, and 0.9 differed between gains and losses (all *BF* > 32.86) and support that the weighting of probability differed for probability 0.3 (*BF* = 8.05) and inconclusive evidence for probability 0.1 (*BF* = 1.28).

The medians of the individual estimates of the Prelec (1998) two‐parameter probability weighting function were *γ*^+^ = 0.46 and *δ*^+^ = 1.04 for risk and gains, *γ*^+^ = 0.39 and *δ*^+^ = 0.95 for ambiguity and gains, *γ*^−^ = 0.64 and *δ*^−^ = 0.85 for risk and losses, and *γ*^−^ = 0.54 and *δ*^−^ = 0.66 for ambiguity and losses. The estimates for gains and risk are very close to the ones observed by Bleichrodt and Pinto (2000) for health. The difference in event weighting between risk and ambiguity was due to a difference in the parameter *δ*^−^, reflecting pessimism (*BF* = 5.46). For the other parameters (*γ*^+^, *γ*^−^, and *δ*^+^), we found support for the null that they were the same for risk and ambiguity (all *BF* < .33). Wakker (2010) has argued that likelihood insensitivity reflects the cognitive component of event weighting and pessimism the motivational component. Our data thus suggest that the observed ambiguity aversion for mixed health prospects was caused by motivational factors.

## 5 DISCUSSION

We have completely measured ambiguity preferences for health. We started with a general model that includes many of the ambiguity models that have been proposed in the literature as special cases. We showed how the different parameters of this general model (utility, loss aversion, and event weighting) could be measured. This made it possible to address the two central questions of this paper: To what extent do health preferences under ambiguity differ from those under risk, and if so, how can we best model these ambiguity preferences? By answering these questions, we have gained insight into the question whether and to what extent we can use findings from the rich literature on health decision under risk to inform health decisions under ambiguity where evidence is thin on the ground. Moreover, we have drawn some conclusions about the descriptive validity of the large set of ambiguity models that have been proposed.

Our data suggest that many of the results that have been derived for health decision making under risk may carry over to ambiguity. We found support that utility, loss aversion, and event weighting for gains are the same between risk and ambiguity. We only observed a difference in event weighting for losses. Consequently, results derived under risk and involving only gains may prove to be useful in predicting preferences under ambiguity. This is an important conclusion for health technology assessments where decisions are typically made under ambiguity but measurement methods use risk (such as in the standard gamble). Our results suggest that the bias resulting from this simplification may be small for gains. On the other hand, the distinction between health gains and health losses is important as our subjects treated gains and losses differently and they were more pessimistic under ambiguity in the loss domain. Our results, therefore, also indicate that assuming that patients behave according to expected utility, an assumption that is frequently made in health technology assessment, is too restrictive and may well lead to biased recommendations. Typically, (medical) decision analysts assume that patients consider all uncertainty as equivalent and reducible to a probability distribution such as the one the physician provides (Curley et al., 1984). The observed difference between risk and ambiguity for health losses suggests that patients value reductions in the clinical ambiguity of losses, but less so in gains. Ignoring this finding may lead to treatment recommendations that do not properly reflect patients' preferences.

Regarding the descriptive validity of ambiguity models, our data provide support for models such as prospect theory that capture ambiguity aversion through a difference between probability and event weights. Theoretical approaches in health economics have usually adopted the smooth model, the most prominent SDU model. Our results suggest that this may not be the best approach to describe people's preferences for health. Performing theoretical analyses using prospect theory is more complex than analyses based on the smooth model, but this additional complexity may be worth the price if the aim is to accurately describe people's preferences.

The difference between probability weights and event weights for losses could explain the ambiguity aversion for mixed prospects that we observed. The absence of differences in utility, event weighting, and loss aversion for health gains is consistent with the absence of ambiguity aversion for health gains in our study. This absence may be surprising. On the other hand, evidence from money also suggests that ambiguity aversion may not always be the dominant pattern (e.g., Binmore, Stewart, & Voorhoeve, 2012; Charness, Karni, & Levin, 2013; Kocher et al., 2018). Our findings suggest that the effects of ambiguity in health will be most pronounced for losses. Perhaps unexpectedly, our results predict ambiguity aversion for health losses whereas the literature on money suggests that ambiguity seeking prevails for losses. However, the strongest deviation between event weights and probability weights occurred for unlikely losses and for these usually more ambiguity aversion is observed (Trautmann and van de Kuilen, 2015).

VoI analysis has become popular in health technology assessment to value the expected gain from reducing uncertainty through the collection of more data. VoI is based on Bayesian principles and has typically not considered ambiguity aversion, which implies a deviation from Bayesianism. In a nonexpected utility framework (assuming an SDU model), Snow (2010) showed that the VoI increases with ambiguity aversion. Our data thus suggest that the VoI is higher when information about health losses is provided than when information about health gains is provided. In interpreting these results and assessing the extent to which VoI analyses should take them into account, it is important to keep in mind that our results are descriptive, although VoI analyses often also have a prescriptive orientation.

We made several assumptions throughout our analysis. The assumption of biseparable preferences seems reasonable. As mentioned above, biseparable preferences are very general and the data of Abdellaoui et al. (2016) support the general assumption underlying biseparable preferences for money.

A crucial assumption is that subjects take 50 years as their reference point. We induced this reference‐dependent thinking by coding all outcomes as gains and losses from 50 years. The results of Attema et al. (2013) provide support for our way of inducing subjects to adopt this reference point, but it would be desirable to know more about the reference point that subjects adopt in experiments about health.

We followed Curley et al. (1984) by implementing ambiguity through the specification of ranges of possible probabilities. This implementation is still close to risk as a range of probabilities is given. It might be that this closeness has to some extent been responsible for the observed similarity of the results for risk and ambiguity (except event weighting for losses). Baillon, Cabantous, and Wakker (2012) show that different ways of implementing ambiguity lead to different results. One interesting avenue to explore is to introduce events for which no probabilistic information is given at all (e.g., by using vague terms such as likely and unlikely), to measure the subjects' beliefs about these events, for example, using the method of Baillon (2008), and to explore whether the results of our paper can be replicated in this context, which arguably may be closer to medical practice.

Another possible extension would be to use a different reference point than 50 years, as we did. Arguably, 50 years is quite high and perhaps subjects' ambiguity preferences would change if the induced reference point would be less than 50 years. On the other hand, a reference point of 50 years is close to subjects' actual life expectancy and this may have made it easier for them to adopt. Lower reference points might not be perceived as neutral but as a loss, and thus, in those cases, our method for inducing the reference point might be less successful.

## 6 CONCLUSION

Many medical decisions involve ambiguity. Empirical research suggests that people are not neutral towards ambiguity, but health economics research has typically ignored ambiguity attitudes. This paper has explored ambiguity attitudes for health, and we have in particular concentrated on the question how they can best be modeled. We assumed a general model of ambiguity preferences and measured its different components for risk and ambiguity. For health gains, we found no differences between risk and ambiguity suggesting that in this domain, we can use the rich literature on health decision making for risk to inform health decision making under ambiguity. For health losses, however, we found a difference in event weighting between risk and ambiguity. Utility and loss aversion were the same. Our data provide support for models such as prospect theory that explain ambiguity attitudes through a difference in event weighting. Utility was convex for losses and linear to concave for gains. Event weighting was inverse S‐shape reflecting the overweighting of unlikely events and the underweighting of more likely events. Finally, we found support for loss aversion with health losses weighting about 1.5 times as much as health gains. In conclusion, given that most medical interventions involve losses in life expectancy, our result of different preferences for risky and ambiguous health losses is likely to be very relevant for medical decision making.

## Supporting information

Data S1. Online Appendix. Measuring Ambiguity Preferences for Health
