# Supplementary material for: Ambiguity preferences for health
Source: Health Econ. 2018 Jul 3;27(11):1699–716. doi: 10.1002/hec.3795 (PMC6221042; doi:10.1002/hec.3795)
Supplement: Supplementary file 1 — Data S1. Online Appendix. Measuring Ambiguity Preferences for Health [file HEC-27-1699-s001.pdf]

Measuring Ambiguity Preferences for Health

Online Appendix

June 19, 2017

1 Non parametric tests

1.1 Consistency checks

Table 1 show the results of the comparison between the original and repeated answers of x_3^+ for both risk and ambiguity.

	Risk	Ambiguity
wilcoxon test (p-value)	0.01	0.00
Bayes factor	1.25	4.34

Table 1: Statistical tests for repeated elicitation of x_3^+

1.2 Ambiguity aversion

Table 2 shows the results of the comparison ($L_u > L_r$ for mixed prospects and $x_{1r}^+ > x_{1u}^+$ for gain prospects).

	L	x_1^+
wilcoxon test (p-value)	0.00	0.43
Bayes factor	2.92	0.14

Table 2: Statistical tests (one-sided tests) for ambiguity aversion on L and x_1^+

1.3 The utility for gains and losses

Table 3 shows the results of statistical tests on the comparison between areas under the normalized utility function and 0.5 (the case of linear utility).

1.4 Loss Aversion

Table 4 shows the value of the significance tests for the comparison between loss aversion coefficient and 1 (one-sided tests).

	Risk, gains	Risk, losses	Ambiguity, gains	Ambiguity, losses
wilcoxon test (p-value)	0.04	0.00	0.58	0.00
Bayes factor	0.95	5.67	0.14	370.22

Table 3: Statistical tests on the comparison between area under the normalized utility function and 0.5

	mean risk	median risk	mean unc	median unc
Wilcoxon test	0.00	0.00	0.00	0.00
Bayes factor	15.22	13.02	12.34	7.39

Table 4: Loss aversion coefficients, Kahneman and Tversky definition

Table 5 shows the results of the statistical tests on the difference between risk and ambiguity for the mean and median loss aversion coefficients (columns (1) and (2) correspond to Kahneman and Tversky definition for the mean and median coefficients).

	KT mean	KT median	Kobb. and Wakker
wilcoxon test	0.40	0.44	0.26
Bayes factor	0.14	0.14	0.17

Table 5: Loss aversion coefficients, Kahneman and Tversky definition, difference between risk and ambiguity

1.5 Probability weighting and event weighting

1.5.1 Risk vs. ambiguity

Table 6 shows the results of statistical tests on the difference between risk and ambiguity for gains.Table 7 shows the results of statistical tests on the difference between risk and ambiguity for losses.

	$p = 0.1$	$p = 0.3$	$p = 0.5$	$p = 0.7$	$p = 0.9$
wilcoxon test	0.51	0.45	0.04	0.48	0.13
Bayes factor	0.14	0.14	1.09	0.19	0.56

Table 6: Comparison of decision weights under risk and ambiguity, gains

	$p = 0.1$	$p = 0.3$	$p = 0.5$	$p = 0.7$	$p = 0.9$
wilcoxon test	0.00	0.01	0.16	0.38	0.55
Bayes factor	45.14	4.12	0.93	0.20	0.14

Table 7: Comparison of decision weights under risk and ambiguity, losses

1.5.2 Overweighting and underweighting

Table 8 show the results of the statistical tests on the difference between decision weights and probabilities (or the center of the probability interval under ambiguity).

	$p = 0.1$	$p = 0.3$	$p = 0.5$	$p = 0.7$	$p = 0.9$
Risk, gains, wilcoxon test	0.00	0.06	0.17	0.00	0.00
Risk, gains, Bayes factor	1047621.47	6.11	0.30	6811.77	264464154.09
Amb., gains, wilcoxon test	0.00	0.10	0.00	0.00	0.00
Amb., gains, Bayes factor	33951025.45	2.13	42.50	73932.35	25915534666.01
Risk, losses, wilcoxon test	0.00	0.00	0.23	0.12	0.00
Risk, losses, Bayes factor	991518.05	93.83	0.21	1.14	69295.44
Amb., losses, wilcoxon test	0.00	0.00	0.00	0.40	0.00
Amb., losses, Bayes factor	96786466669.81	7081750.18	32.44	0.28	9260222.98

Table 8: Decision weights under risk, gains. Statistical tests

1.5.3 Gains vs. losses

Table 9 shows the results of statistical tests on the difference between gains and losses under risk and ambiguity.

	$p = 0.1$	$p = 0.3$	$p = 0.5$	$p = 0.7$	$p = 0.9$
Risk, wilcoxon test	0.76	0.61	0.09	0.01	0.01
Risk, Bayes factor	0.14	0.15	0.46	4.95	5.82
Amb., wilcoxon test	0.05	0.01	0.00	0.00	0.00
Amb., Bayes factor	1.28	8.04	1052.35	32.86	67.18

Table 9: Comparison of decision weights between gains and losses. Statistical tests

1.5.4 Parametric weighting functions

Table 10 shows the results of the statistical tests of the comparison between parameters δ^s and γ^s and 1, for $s = +/-$.

	risk, gains	risk, losses	ambiguity, gains	ambiguity, losses
δ, Wilcoxon test	0.00	0.00	0.00	0.00
δ, Bayes factor	82167.29	0.26	733401875451.44	0.14
γ, Wilcoxon test	0.99	0.01	0.82	0.00
γ, Bayes factor	0.14	1.98	0.29	1595561.28

Table 10: Significance of weighting function parameters

Table 11 shows the comparison between estimated parameters between gains and losses and risk and ambiguity.

	Risk: G/L	Unc.: G/L	Gains: R/U	Losses: R/U
δ, Wilcoxon test	0.01	0.09	0.14	0.03
δ, Bayes factor	0.60	0.27	0.33	0.16
γ, Wilcoxon test	0.10	0.00	0.51	0.01
γ, Bayes factor	0.65	2.78	0.31	5.46

Table 11: Weighting function parameters, statistical tests. G/L: gains vs. losses; R/U: risk vs. ambiguity

2 Parametric estimation of utility

2.1 Estimation on median data

Table 12 shows the values of the utility function (power family) estimated on the median data.

	Risk, gains	Risk, losses	Ambiguity, gains	Ambiguity, losses
Coefficient	0.91	0.76	0.99	0.69
standard error	0.02	0.04	0.02	0.04

Table 12: Estimation of utility function (power) on median data

2.2 Individual estimations

Table 13 shows the results of the parametric estimations of the utility function at the individual level.

	Risk, gains	Risk, losses	Ambiguity, gains	Ambiguity, losses
median	0.89	0.88	0.95	0.80
Q25	0.66	0.67	0.79	0.59
Q75	1.21	1.04	1.20	1.01
wilcoxon test	0.20	0.01	0.93	0.00
Bayes factor	0.18	0.15	0.42	0.60

Table 13: Summary statistics for individual utility parameters

Table 13 also shows the results of the statistical test on the comparison between the elicited values and 1, the case for linear utility.

Table 14 shows the results of statistical tests on the difference between gains and losses, and between risk and ambiguity.

	Risk: G/L	Unc.: G/L	Gains: R/U	Losses: R/U
wilcoxon test (p-value)	0.95	0.01	0.14	0.10
Bayes factor	0.14	0.80	0.45	0.25

Table 14: Statistical tests on the comparison between utility parameters

Table 15 shows the results of ANOVA on utility parameters and confirms the absence of strong difference in utility parameters between domain or contexts.

	Bayes Factor	standard anova, Pr[>F]
domain + subj	1.280	0.038
context + subj	0.140	0.626
domain:context + subj	0.892	0.141

Table 15: ANOVAs for utility parameters

3 Kobberling and Wakker (2005) definition of loss aversion

Table 16 shows the summary statistics of loss aversion coefficients according to Kobberling and Wakker (2005) definition. Table 16 also shows the statistical tests for the comparison with 1 (one-sided tests). Loss aversion was significant in both cases.

Table 17 shows the classification of individual based on their loss averse behavior, with most subjects loss averse unde risk and under ambiguity.

	Risk	Ambiguity
median	1.46	1.75
q25	1.00	1.00
q75	2.14	2.75
Wilcoxon test	0.00	0.00
Bayes factor	10.44	44.12

Table 16: Loss aversion coefficients, Kobberling and Wakker definition

	loss averse	gain seeking	neutral
risk	47	15	3
ambiguity	48	14	3

Table 17: Classification of loss averse behavior, Kobberling and Wakker definition

Table 18 shows the statistical tests on the difference between the two definitions of loss aversion (for risk and ambiguity). Results show that both definitions gave similar results.

	Risk (1)	Ambiguity(1)	Risk(2)	Ambiguity(2)
wilcoxon test	0.45	0.16	0.59	0.24
Bayes factor	0.18	0.30	0.15	0.29

Table 18: Difference between elicited loss aversion coefficients, Kahneman and Tversky definition ((1): median, (2): mean) vs. Kobberling and Wakker definition

4 Neo-additive weighting functions

We estimated the neo-additive decision weighting function defined as:

$$w(p) = (1 - a) * p + (a - b)/2$$

where p is a probability measure. For a given value of a, increases in b shifts the decision weight downward. Parameter b can be interpreted as a measure of pessimsim. Higher values of b indicate more pessimism and negative values of b indicate optimism. Parameter a measures the weight given to changes in likelihoods For a given value of b, increases in a decrease the slope of the weighting function and the decision maker is less sensitive to probability changes. There a parameter a can be interpreted as a measure of likelihood insensitivity. If $a = 0$ and $b = 0$, the decision weights is equal to probability p.

Table 19 shows the results of the estimation of parameters a^s and b^s at the individual level, for $s = +/-$.

	risk, gains	risk, losses	ambiguity, gains	ambiguity, losses
median a	0.48	0.34	0.55	0.53
q25 a	0.27	0.16	0.33	0.33
q75 a	0.68	0.57	0.83	0.75
median b	0.07	-0.01	0.11	-0.15
q25 b	-0.20	-0.22	-0.09	-0.32
q75 b	0.30	0.14	0.28	0.06

Table 19: Linear weighting function parameters a^s and b^s, $s = +/-$

Table 20 shows the results of the statistical tests of the comparison between parameters a and b and 0.

	risk, gains	risk, losses	ambiguity, gains	ambiguity, losses
a, Wilcoxon test	0.0000000000E+00	0.0000000000E+00	0.0000000000E+00	0.0000000000E+00
a, Bayes factor	4.1733589192E+14	3.8354619640E+11	2.4754456474E+19	1.9032103191E+18
b, Wilcoxon test	1.5400000000E-01	3.2400000000E-01	1.4000000000E-02	0.0000000000E+00
b, Bayes factor	3.1900000000E-01	2.5000000000E-01	3.3350000000E+00	2.3145600000E+02

Table 20: Significance of weighting function parameters

Table 21 shows the comparison between estimated parameters between gains and losses and risk and ambiguity.

	Risk: G/L	Unc.: G/L	Gains: R/U	Losses: R/U
a, Wilcoxon test	0.01	0.12	0.18	0.00
a, Bayes factor	6.17	0.30	0.37	127.62
b, Wilcoxon test	0.05	0.00	0.23	0.01
b, Bayes factor	0.73	536.55	0.36	3.00

Table 21: Weighting function parameters, statistical tests. G/L: gains vs. losses; R/U: risk vs. ambiguity

.

.

5 Experimental instructions

Welcome to this experiment on decision-making regarding medical treatment.

The experiment consists of several parts. First we ask you to read the instructions. To find out whether you have understood these instructions, we start with a couple of practice questions. Take your time to read the instructions and consider the questions carefully. If you have any questions or remarks, don't hesitate to consult the experimenter.
We would like to emphasize that we are only interested in your preference, so there are no right or wrong answers.

Context

Suppose you carry a disease which does not lower your quality of life, but does lower your life expectancy (for instance diabetes, which is known not to cause worse health when one takes their drug, but will cause premature death). In particular, imagine that you can expect to live 50 more years because of this disease. You need a drug to survive though. Therefore, you are currently taking Drug A, which has no side-effects.

An example of decisions you will have to make

Recently, the doctors have developed a new drug (Drug B) that can do something about your disease (and, hence, your life expectancy). However, the drug is risky and can have two different outcomes: there is a 50% probability (Issue 1) that the drug will work well and will cause a gain in life expectancy, but there is also a 50% probability (Issue 2) that the drug will not work well and then it will actually reduce life expectancy. It is not possible to determine in advance what the drug's outcome will be. This will only be resolved once the drug has been taken. In the following questions please indicate whether you would take Drug B or stick with Drug A.

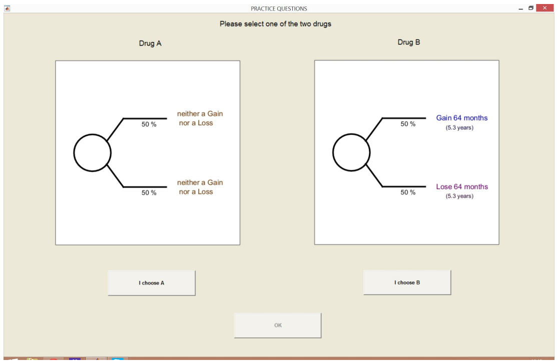

Once you'll have given your preference. You will face a similar question, except that one amount of Drug B has changed. Please indicate whether you would take Drug B or stick with Drug A.

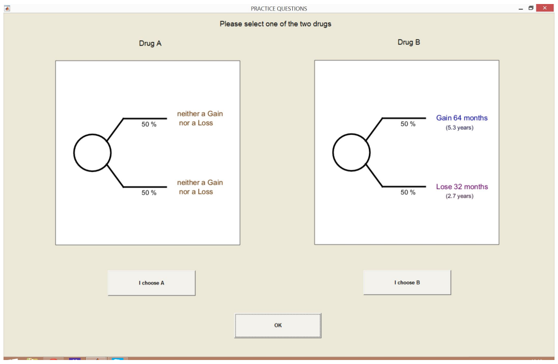

The program will continue to ask you similar questions until it gets a precise idea of your switching point between Drug A and Drug B. For each of these questions, once you have selected your preferred alternative, click on OK to proceed.

When the program gets a sufficiently precise idea of your switching point between the two drugs, you will be able to proceed to the next question. If you want to reconsider your previous choices, you will be able to Cancel them and start anew.

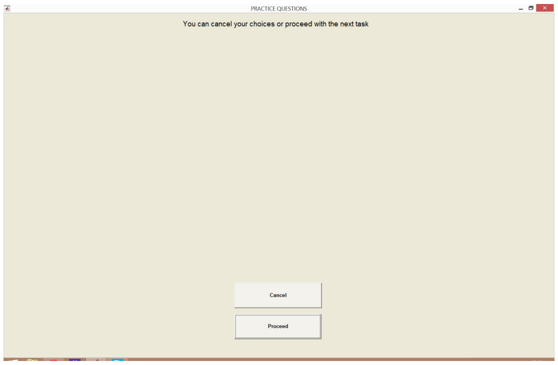

A second example of decisions you will have to take

The experiment consists of several parts. You will face different types of decisions. For some decisions, the treatments will be documented and you will have access to the exact chances (in %) associated with each outcome. For some decisions, the treatments will be undocumented and you won't have access to such precision information: you will only know a probability range for each outcome. For example, the decision shown above becomes, in the undocumented treatment, the following decision:

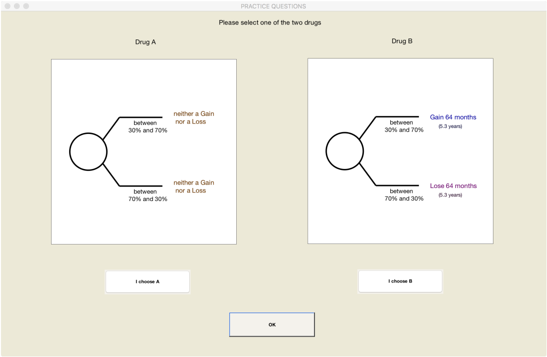

Note that a 30% chance for the upper branch corresponds to a 70% chance for the lower branch. Similarly, a 70% chance for the upper branch corresponds to a 30% chance for the lower branch.

For some other decisions, the two drugs are risky and can have two different outcomes. For example, both drugs have a probability (or a probability range in the undocumented treatment) that the drug will work well and will cause a gain in life expectancy, but they also have a probability (or a probability range) that the drug will not work well and then it will not increase or actually reduce life expectancy.

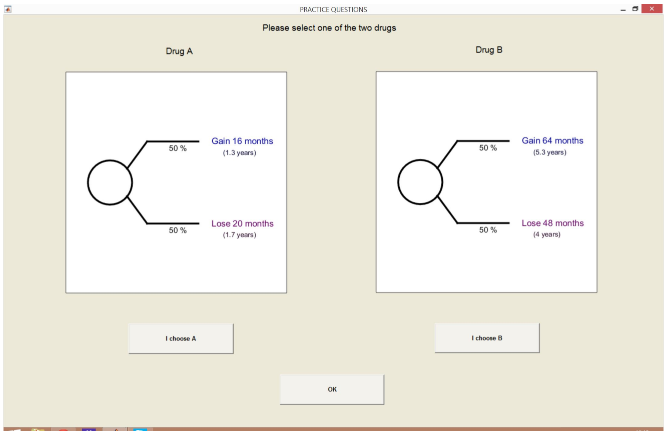

With Drug B, the amount of the gain is higher than with Drug A, but the amount of the loss is also higher with Drug B than with Drug A. It is not possible to determine in advance what the drug's outcome will be. This will only be resolved once the drug has been taken. In the following questions please indicate whether you would choose Drug A or Drug B.

A third example of decisions you will have to take

Please now consider the following situation. This is the same situation as in the beginning of the experiment, but with the difference that the doctors have now developed two new drugs (instead of one) that can do something about your disease (and, hence, your life expectancy). One drug (Drug A) is risky. With 50% probability (or a probability range in the undocumented treatment), it gives a high gain in life expectancy, but it also has a 50% probability (or a probability range in the undocumented treatment) to be less effective, in which case it gives a smaller gain in life expectancy. Drug B gives a certain improvement of a specified duration, which is smaller than the best outcome of Drug A, but higher than the worst outcome of Drug A. Please indicate which of the two drugs you would prefer for each of the given situations.

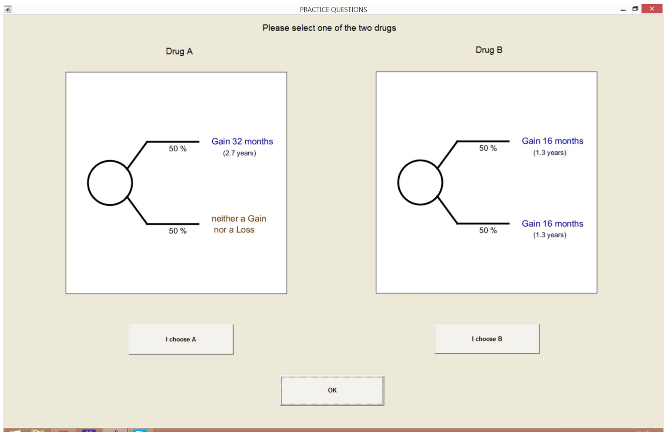

Just as above, the program will continue to ask you similar questions until it gets a precise idea of your switching point between Drug A and Drug B.

For each set of decisions, pay attention not only to the amounts but also to the chances.

A last example of the decisions you will have to take

Please now consider the following situation, which is the same as the previous one, but with the difference that you now have to imagine to have a more aggressive form of the disease. The disease is such that you cannot stick to your current drug or you would lose a very large amount of life expectancy compared to people with the common, milder form of the disease. However, the doctors have developed two drugs for this more aggressive form that can do something about your loss in life expectancy. The outcome of one of these drugs (Drug A) is risky. With 50% probability (or a probability range in the undocumented treatment), this drug works well, causing your life expectancy to stay the same as for patients with the mild form of the disease (that is, 50 years). It also has a 50% probability (or a probability range in the undocumented treatment) not to work well, and then you will lose some lifetime compared to patients with the mild form. Drug B gives a sure outcome, which will be a loss compared to the mild form patients, but the amount of the loss will be lower than in case of Issue 2 of Drug A. Please indicate which of the two drugs you would prefer for each of the given situations. Note that your loss will always be higher if you stick to your old drug, so you have to choose one of Drugs A and B.

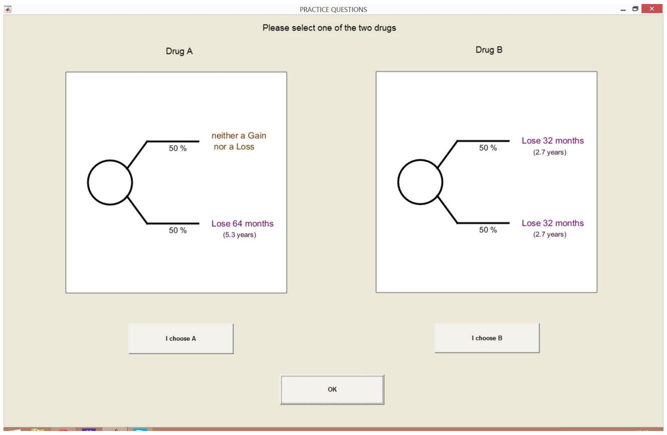

Just as above, the program will continue to ask you similar questions until it gets a precise idea of your switching point between Drug A and Drug B. For each set of decisions, pay attention not only to the amounts but also to the chances: for some choice situations, the chances associated with Issue 1 and Issue 2 can be different from 50/50% chances.
